# Brincidofovir Use after Foscarnet Crystal Nephropathy in a Kidney Transplant Recipient with Multiresistant Cytomegalovirus Infection

**DOI:** 10.1155/2017/3624146

**Published:** 2017-02-27

**Authors:** Romain Vial, Christine Zandotti, Sophie Alain, Alexandre Decourt, Noémie Jourde-Chiche, Raj Purgus, Charleric Bornet, Laurent Daniel, Valérie Moal, Tristan Legris

**Affiliations:** ^1^Centre de Néphrologie et Transplantation Rénale, Hôpital Conception, Assistance Publique-Hôpitaux de Marseille, Marseille, France; ^2^Aix-Marseille University, Marseille, France; ^3^Fédération de Bactériologie-Virologie-Hygiène, Hôpital Timone, Assistance Publique-Hôpitaux de Marseille, Marseille, France; ^4^Laboratoire de Bactériologie-Virologie-Hygiène, Centre Hospitalier Universitaire, Limoges, France; ^5^Pharmacie Hospitalière, Hôpital Conception, Assistance Publique-Hôpitaux de Marseille, Marseille, France; ^6^Laboratoire d'Anatomie Pathologique, Hôpital Timone, Assistance Publique-Hôpitaux de Marseille, Marseille, France

## Abstract

*Background*. Cytomegalovirus (CMV) antiviral drug resistance constitutes an increasing challenge in transplantation. Foscarnet is usually proposed when resistance for ganciclovir is suspected, but its use is limited by its nephrotoxicity.* Case Presentation*. We report a case of multiresistant CMV disease in a kidney transplant recipient. Foscarnet was prescribed after ganciclovir treatment failure in a patient with two mutations in the UL97 viral gene. Foscarnet induced biopsy-proven kidney crystal precipitation that resulted in severe acute transplant failure and nephrotic syndrome. Despite a large decrease in immunosuppression, CMV disease was not controlled and a salvage therapy with Brincidofovir (BCV), which is an oral lipid conjugate of cidofovir with limited nephrotoxicity, was attempted. Clinical and virological remission was observed after a 21-day course of BCV, despite mild and reversible liver toxicity. However, a new relapse could not be effectively cured by BCV due to a new mutation in the UL54 gene, which is known to confer resistance to cidofovir. A new course of foscarnet finally resulted in prolonged CMV remission. Herein, we present a review of foscarnet nephropathy cases in solid-organ transplanted patients.* Conclusions*. This unique case highlights the potential benefit of BCV use during resistant CMV infection, although mutations in the UL54 gene may limit its therapeutic efficacy. These findings need to be confirmed in clinical trials.

## 1. Introduction

Human cytomegalovirus (CMV) infections represent a major clinical issue after solid-organ transplantation, especially when the donor's serological status is positive and the recipient is seronegative (D+/R−) [1]. Despite improvement in diagnosis, prevention, and treatment strategies [2–4], concerns related to antiviral drug resistance (ADR) constitute an increasing challenge for the transplant physician. ADR was tested in a retrospective French cohort study of D+/R− kidney transplant recipients up to one year after transplant if the viremia increased during antiviral therapy. ADR was observed in 16% of the 80 patients who were treated preemptively versus 3% of the 32 patients who received 3 months of valganciclovir-based prophylaxis (*p* = 0.05) [5]. Other risk factors for ADR include the type of transplant (highest risk for lung and kidney-pancreas recipients), D+/R− serostatus, delay in commencement of prophylaxis, high peak blood viral load (>10^5^ copies/ml), increased duration of antiviral exposure, and suboptimal drug concentration [6]. Intravenous ganciclovir (GCV) or oral valganciclovir (vGCV) is recommended as the first-line treatments for CMV disease within the transplant population [2]. Other drugs, such as foscarnet (FOS) and cidofovir (CDV), are typically proposed as second-line therapies when ADR is suspected. However, FOS and CDV cause significant nephrotoxicity, and FOS also induces electrolyte abnormalities. Brincidofovir (BCV), which is a new orally bioavailable lipid acyclic nucleoside phosphonate that is converted intracellularly into CDV diphosphate, has limited renal toxicity [7]. Here, we report a case of BCV use in the setting of FOS nephrotoxicity in a transplant recipient with CMV ADR.

## 2. Case Presentation

A 42-year-old woman with stage 5D chronic kidney disease related to MYH9-related disease received her first kidney transplant from a familial living donor. Repeated anti-HLA antibody blood testing was negative, despite previous blood transfusions and pregnancies. The A, B, DR, and DQ HLA mismatches were 0/1/1/1, respectively. The CMV serostatus was D+/R−. Her immunosuppressive regimen consisted of an induction with antithymocyte globulins and a maintenance regimen with ciclosporin A, azathioprine, and steroids. She also received cotrimoxazole and vGCV prophylaxis for 6 months (900 mg/d). With the exception of two deep venous thrombosis episodes, the first seven months after transplantation was unremarkable with good transplant function (serum creatinine level = 100 *μ*mol/L). One month after vGCV discontinuation on day (D) 222 after graft, she was admitted for a fever of 38.2°C and epigastric pain without diarrhea. Her biological tests revealed a severe lymphocytopenia (150/mm^3^). Her serum creatinine level was 122 *μ*mol/L. The blood and urine cultures and liver and pancreatic tests were unremarkable, whereas her pp65 antigenemia was strongly positive (600 positive cells/200000 polymorphonuclear leukocytes (PC)). The chest X-ray and fundus examination did not suggest pulmonary or retinal CMV involvement. An upper gastrointestinal endoscopy revealed gastritis with a positive CMV polymerase chain reaction (PCR). Due to the diagnosis of possible CMV gastrointestinal disease [4], a first intravenous GCV treatment (10 mg/kg/d) was started together with a decrease in immunosuppression (half azathioprine dose), resulting in a slow but sustained decrease in the CMV antigenemia. Two months of GCV was needed to obtain viral clearance defined by two consecutive weekly negative antigenemia results.

**Figure 1:**
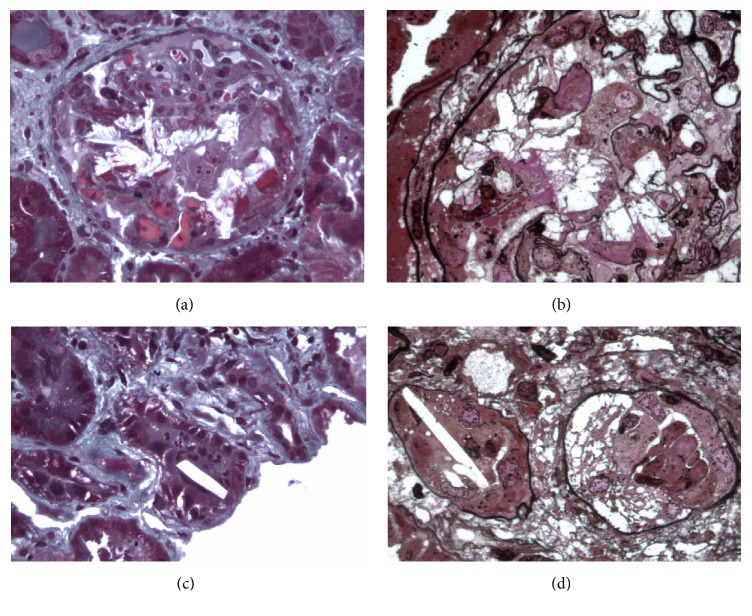
Foscarnet nephropathy in the kidney transplant. (a) Masson's trichrome staining (×200) showed intraglomerular crystalline precipitation obstructing the capillaries and crushing the mesangium together with fibrinoid thrombi. (b) Jones methenamine silver staining (×400) also revealed FOS crystals within the glomerular capillaries. (c) Masson's trichrome staining (×200) showed crystals that resembled short sticks with angular edges in the tubular lumen. (d) Jones methenamine silver staining (×400) also revealed FOS crystals within the tubular lumen.

One week after GCV discontinuation on D288, she was readmitted for fever recurrence and abdominal pain and a relapse of CMV disease was observed (antigenemia = 20 PC). No retinitis was found again. As digestive symptoms were similar to recent primary CMV infection, no new upper endoscopy was performed. A new GCV-based treatment (10 mg/kg/day) was started. No clinical or biological improvement was noted after two weeks of treatment (antigenemia = 115 PC) despite azathioprine withdrawal. Genotypic testing for ADR in the UL97 kinase gene revealed two common mutations (A594V and L595S) associated with moderate viral resistance for GCV [13] and no mutation in the UL54 DNA polymerase gene. The kidney graft function was stable (serum creatinine level = 100 *μ*mol/L). Thus, a second-line treatment with intravenous FOS (180 mg/kg/d) was initiated at D306. A fast decrease in the CMV viral load was observed that allowed FOS weaning at D320. A second relapse of CMV infection (same symptoms, antigenemia = 25 PC, blood quantitative CMV PCR = 351000 DNA copies/mL) at D329 led us to restart FOS at the same dose. Unfortunately, 6 days after this new FOS course a severe acute graft failure was noted (serum creatinine level = 450 *μ*mol/L, blood ciclosporin trough level = 200 ng/mL) that was associated with an acute nephrotic syndrome (urinary protein creatinine ratio = 4.5 g/g and serum albumin = 2.6 g/dL). A transplant biopsy showed diffuse tubular necrosis and tubular and intraglomerular crystal deposits that obstructed capillaries and were suggestive of FOS nephropathy. No sign of acute rejection or CMV transplant infection was noted on the biopsy (Figure 1). At that time, the CMV antigenemia had become negative, blood CMV PCR was weak (1381 copies/mL), and FOS was stopped, which allowed a partial reversal of the glomerulopathy and transplant failure (serum creatinine level = 150 *μ*mol/L and proteinuria = 1 g/d).

**Figure 2:**
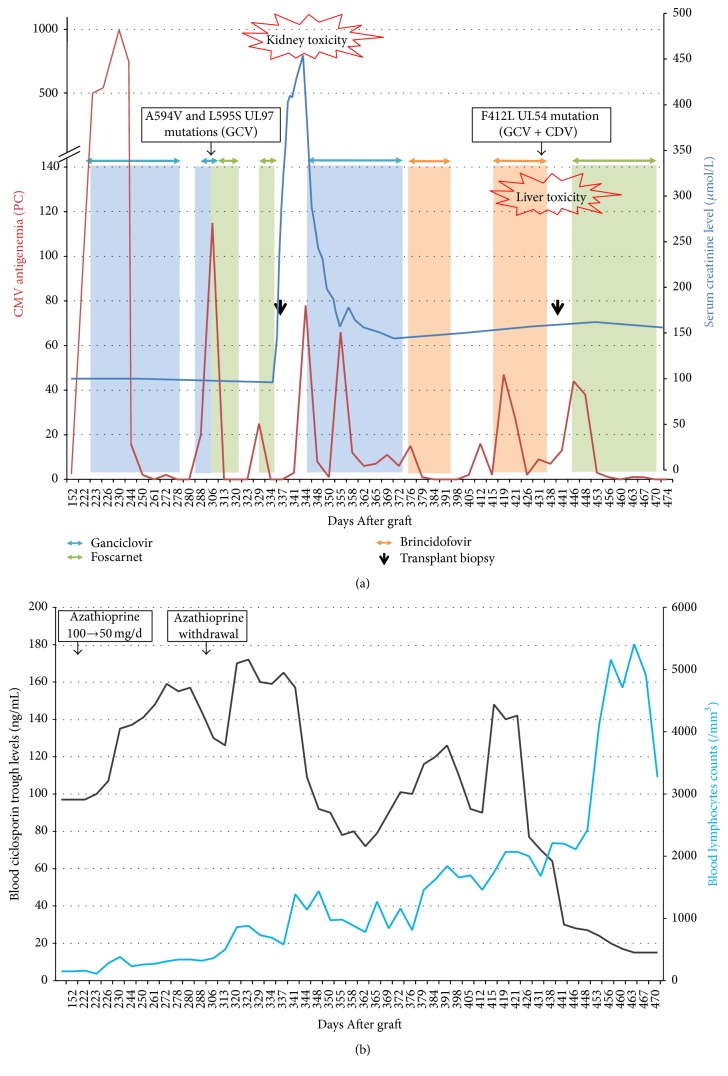
Course of multiresistant CMV disease. (a) Evolution of CMV antigenemia (red line) and kidney transplant function (serum creatinine level, blue line) together with antiviral therapies and genotypic mutations. PC: positive cells/200000 polymorphonuclear leukocytes. GCV: ganciclovir. CDV: cidofovir. (b) Evolution of blood ciclosporin levels (black line) and lymphocytes counts (blue line).

However, at D341 a third CMV relapse occurred (antigenemia = 78 PC, PCR = 141000 copies/mL). Because the FOS toxicity was recent, we decided to begin a double-dose of GCV therapy [14] together with intravenous immunoglobulins (2 infusions of 0.3 g/kg/d at a 10-day interval) and a decrease in the ciclosporin target trough level (100 ng/mL). One month later, persistent low-grade symptoms and mild but positive CMV antigenemia (5–15 PC) reflected the failure of this regimen. A second genotypic testing for ADR revealed the same L595S mutation in the UL97 gene without mutation in the UL54 gene. To avoid new FOS toxicity, oral BCV was introduced (100 mg orally twice a week) at D376 for 21 days with good results on clinical and viral parameters (negative antigenemia and blood PCR after 17 days). A mild isolated cytolysis (alanine and aspartate aminotransferase (ALT and AST) = 137 and 104 IU/L, respectively) and moderate epigastric pain were observed during therapy. Liver ultrasonography was normal. The liver enzymes returned to normal levels and the abdominal pain disappeared after BCV discontinuation. Nevertheless, at D412 a 4th relapse was observed (antigenemia = 16 PC, blood PCR = 95700 copies/mL) and was again treated with BCV for 15 days (200 mg/week). After 15 days of BCV, persistent diarrhea and abdominal pain indicated a new upper gastrointestinal endoscopy together with a colonoscopy. CMV PCR biopsies of stomach and large bowel were positive (3380 and 3020 copies/mL, respectively). Blood CMV antigenemia and PCR were 9 PC and 38200 copies/mL, respectively, after 15 days of BCV. A new mild and isolated cytolysis (ALT = 143, ASAT = 155 IU/L) appearing at the same time led us to definitely stop BCV. Liver enzymes returned to normal levels after BCV discontinuation. No liver biopsy was performed. A third genotypic ADR test, performed at the end of the second course of BCV, revealed a novel F412L mutation in the UL54 DNA polymerase gene that was associated with moderate resistance for GCV and CDV but not FOS [15]. Before reinitiating FOS, a new transplant biopsy was performed at D441 (serum creatinine = 150 *μ*mol/L and proteinuria = 1 g/g). Seven of the 25 glomeruli were sclerotic. Interstitial fibrosis and tubular atrophy were mild without crystals, rejection, or CMV signs. A new two-month course of intravenous FOS (50 mg/kg/d, dose tailored to decreased glomerular filtration rate) was necessary to achieve a complete resolution of CMV infection (negative antigenemia and PCR) without any new FOS nephropathy.  Figure 2 describes the course of the CMV disease together with the course of immunosuppression and blood lymphocyte counts.

Currently, the patient's overall condition is normal 6 months after the final treatment, without secondary CMV prophylaxis. Repeated CMV antigenemia tests have been negative, and the graft function remains stable (mean creatinine = 130 *μ*mol/L, proteinuria = 0.6 g/g, without anti-HLA antibodies).

## 3. Discussion

To our knowledge, we report the first published case of refractory CMV infection treated with BCV in a kidney transplant recipient. BCV (formerly CMX001) is a lipid conjugate of CDV and is highly active in vitro against various double-stranded DNA viruses, such as adenoviruses, herpesviruses, human papillomaviruses, polyomaviruses (including BK virus), and orthopoxviruses [7]. BCV can inhibit the UL54 CMV DNA polymerase when it is converted intracellularly into CDV. Interestingly, BCV's in vitro activity is increased by 422-fold compared to CDV [16], probably due to its more efficient cellular uptake facilitated by the lipid chain. In contrast to CDV, which is actively secreted from the blood into kidney proximal tubule cells by organic anion transporters (OAT), BCV is not a substrate of OAT1 and thus has a lower risk of nephrotoxicity.

A phase 2 study of BCV involved 230 CMV-seropositive allogeneic hematopoietic cell transplant (HCT) recipients who were randomized to receive BCV or a placebo to prevent CMV events. The incidence of CMV events was significantly lower among patients who received BCV at a dose of 100 mg twice weekly than among those who received the placebo. Diarrhea, vomiting, and abdominal pain were the most common adverse effects in the group that received this dose. No increased risk of nephrotoxicity was observed [17]. However, in the recent phase 3 SUPPRESS trial, despite an antiviral effect seen at the end of the on-treatment period at week 14 following HCT (with patients who received BCV experiencing fewer clinically significant CMV infections than patients in the placebo group (24 percent versus 38 percent, *p* = 0.002)), the primary endpoint of prevention of significant CMV infection at week 24 was not reached (data reported at the 2016 BMT Tandem Meetings). These clinical results in a population at risk for kidney injuries together with the in vitro findings mentioned above prompted us to try BCV in our difficult case of GCV resistance and previous FOS nephrotoxicity. Since our personal experience, BCV use has been recently reported in other case studies as a potential curative treatment involving severe resistant dsDNA viral infections (CMV, HSV, and VZV) in HCT recipients and immunocompromised cancer patients [18–21].

In our report, BCV treatment resulted initially in a remission of CMV disease but was marked by abdominal pain, diarrhea, and ALT elevation. Liver metabolism has been proposed to be the most likely major route of elimination for BCV [7]. Mild dose-dependent ALT elevations were observed in 10 to 40% of stem-cell recipients [17]. The known metabolic pathway of BCV, the course of our clinical case, the typical damage, and the rechallenging situation are clearly enough to confirm a BCV induced liver damage, even if mild and likely dose-dependent. However, it was difficult to distinguish between side effect of BCV and gastrointestinal CMV infection itself, regarding abdominal pain and diarrhea.

Similar to CDV, CMV resistance to BCV only involves UL54 DNA polymerase mutations and not UL97 mutations [15]. In the phase 2 trial mentioned above, no known resistance-associated mutations were detected in the BCV arms. Two mutations (M827I and R1052C) in the UL54 gene were found in a small number of subjects without decreased susceptibility to BCV, CDV, GCV, or FOS [22]. Our case and another case in a lung transplant recipient treated with BCV [23] illustrated that BCV could be associated with the A987G and F412L UL94 mutations known to confer ADR to CDV.

Thus, BCV could constitute an antiviral alternative in cases with UL97 mutations when FOS is contraindicated or in cases of FOS nephrotoxicity. Foscarnet nephropathy was initially described in the 1980s as a frequent complication in AIDS patients undergoing treatment for CMV infection [24]. In vivo trisodium foscarnet crystals mixed with sodium calcium salt were first identified by infrared microscopy in the glomerular capillary lumens and tubules of AIDS patients [25]. Importantly, isotonic saline infusion of 1.5 to 2.5 liters per day was demonstrated to reduce this renal toxicity by increasing FOS clearance and constituted the best preventive strategy [24]. Nonetheless, renal failure is possible with FOS despite appropriate hydration. Cases of biopsy-proven FOS crystal precipitation in the transplantation field are relatively scarce and are summarized in Table 1. With the exception of one lung recipient, all patients were kidney transplant recipients. FOS nephropathy does not seem to appear during the first days of therapy but rather after several weeks of treatment. Glomerular crystallization seems to be associated with worse acute kidney injury than isolated tubular crystallization [12]. At worst, FOS nephropathy led to kidney graft loss. Interestingly, FOS precipitation was also observed in the lungs, heart, pancreas, and gastrointestinal tract in two patients with severe systemic crystal dissemination [10, 11].

In conclusion, BCV appeared to be useful in this complicated case of CMV resistance. Although CMV became also resistant to BCV and some mild toxicity occurred, the length of BCV course allowed FOS nephropathy to recover, which facilitated repeated use of FOS, ultimate clearance of the virus, and preserved transplant function. A clinical trial of BCV to manage resistant CMV disease is needed.

## Figures and Tables

**Table 1:** Summary of cases of biopsy-proven foscarnet nephropathy during CMV infection in solid organ transplantation.

Reference	Type of transplant	Indication of FOS	Time to nephropathy after FOS initiation	Peak serum creatinine	Proteinuria	Biopsy results	Outcome
[8, 9]	Kidney D+/R−	CMV syndrome Persistent high CMV viremia after 65 days of GCV	30 days (single treatment)	387 *μ*mol/L	7 g/d	Birefringent crystal precipitation surrounded by macrophages in glomeruli and tubules. Fibrinoid thrombi. Black crystals with Von Kossa's reaction.	FOS withdrawn after 27 days. Seven months after FOS nephropathy: creatinine level at 158 *μ*mol/L, proteinuria at 2 g/d. Second transplant biopsy: moderate interstitial fibrosis and tubular atrophy with sclerosis of half glomeruli, no crystal, and positive in situ CMV PCR.

[10]	Lung D−/R+	CMV bronchiolitis M460I mutation in the UL97 gene	35 days (second treatment; first treatment of 4 weeks)	475 *μ*mol/L	NR	Autopsy: birefringent short crystals with angular edges in glomeruli (with rupture of capillaries and Bowman's capsule) and in tubules with tubular necrosis and granulomas.	No FOS withdrawal. Death 5.5 months after CMV disease (mild acute rejection, chronic lung rejection). Crystals found in lungs, esophagus, kidney, epicardium, pericardium, and tricuspid valve at autopsy.

[11]	Kidney D−/R+	Asymptomatic reactivation of GCV resistant strain despite prophylactic valganciclovir	21 days (single treatment)	Anuria, hemodialysis	NR	Crystals in tubules and in one-third of glomeruli with rupture of the basement membrane, tubular necrosis, and macrophages.	Multiorgan (kidney, pancreas, and myocardium) damage due to FOS crystal precipitation. Pancreatitis and myocarditis resolved. Graft loss and dialysis therapy continued.

[12]	Kidney D+/R−	CMV hepatitis and retinitis A594V mutation in the UL97 gene	After 14 days (single treatment)	157 *μ*mol/L	NR	Birefringent crystals in the tubular lumens. Black crystals with Von Kossa's reaction.	FOS withdrawal. Ten months after FOS nephropathy: creatinine level at 112 *μ*mol/L. Second transplant biopsy: disappearance of crystal deposition.

PR	Kidney D+/R−	CMV gastritis A594V and L595S mutations in the UL97 gene	6 days (second treatment; first treatment of 14 days)	450 *μ*mol/L	4.5 g/g of creatininuria	Crystals in glomeruli and tubules.	FOS withdrawal followed by Brincidofovir. Second biopsy: disappearance of crystals, sclerosis of one-third glomeruli, mild interstitial fibrosis, and tubular atrophy. Third treatment with FOS: resolution of CMV disease. One year after FOS nephropathy: creatinine level at 130 *μ*mol/L, proteinuria at 0.6 g/g.

CMV, cytomegalovirus; FOS, foscarnet; GCV, ganciclovir; NR, not reported; PCR, polymerase chain reaction; PR, present report.
